# Intracranial Injury Caused by Transorbital Penetrating Trauma: An E-Scooter Brake Handle as an Unusual Culprit

**DOI:** 10.3390/brainsci15111160

**Published:** 2025-10-29

**Authors:** Paweł Szczurowski, Michał Gontarz, Jarosław Polak, Jakub Bargiel, Krzysztof Gąsiorowski, Kamil Nelke, Grażyna Wyszyńska-Pawelec

**Affiliations:** 1 Department of Cranio-Maxillofacial Surgery, Jagiellonian University Medical College, 30-688 Cracow, Poland; pawel.szczurowski@uj.edu.pl (P.S.); jakub.bargiel@uj.edu.pl (J.B.); krzysztof.gasiorowski@uj.edu.pl (K.G.); grazyna.wyszynska-pawelec@uj.edu.pl (G.W.-P.); 2 Department of Neurosurgery and Neurotraumatology, Jagiellonian University Medical College, 30-688 Cracow, Poland; j.polak@uj.edu.pl; 3 Maxillo-Facial Surgery Ward, EMC Hospital, 54-144 Wrocław, Poland; kamil.nelke@gmail.com

**Keywords:** penetrating injury, brain injury, foreign body, brake handle, transorbital injury, orbital fracture

## Abstract

Transorbital penetrating intracranial injuries are a rare but life-threatening subset of penetrating head traumas. While isolated cases caused by bicycle brake handles have been reported, often with fatal outcomes, this is the first documented case of such an injury caused by an electric scooter (e-scooter) brake handle. The objective is to present the unique management and clinical course of this unusual case. A case of a 76-year-old male is presented. The patient sustained a transorbital intracranial injury after a same-level fall onto a parked e-scooter, which resulted in the brake handle penetrating his left orbit and reaching the third ventricle. A combined maxillofacial and neurosurgical team performed a frontal craniotomy for foreign body removal, followed by duraplasty. No cerebrospinal fluid leakage was detected postoperatively. Imaging and clinical follow-up at six months and one year revealed significant post-traumatic encephalomalacia in the frontal lobes, ventricular enlargement, and persistent neurocognitive deficits, including memory impairment and executive dysfunction. Visual acuity in the affected eye was reduced, with associated orbital fat atrophy and mild ptosis. E-scooter brake handles pose a previously unrecognized risk for severe transorbital penetrating intracranial injuries. This case underscores the critical importance of a multidisciplinary surgical approach to manage complex craniofacial trauma. Despite successful acute management, patients can suffer substantial long-term neurological and functional sequelae, necessitating comprehensive follow-up care.

**Figure 1:**
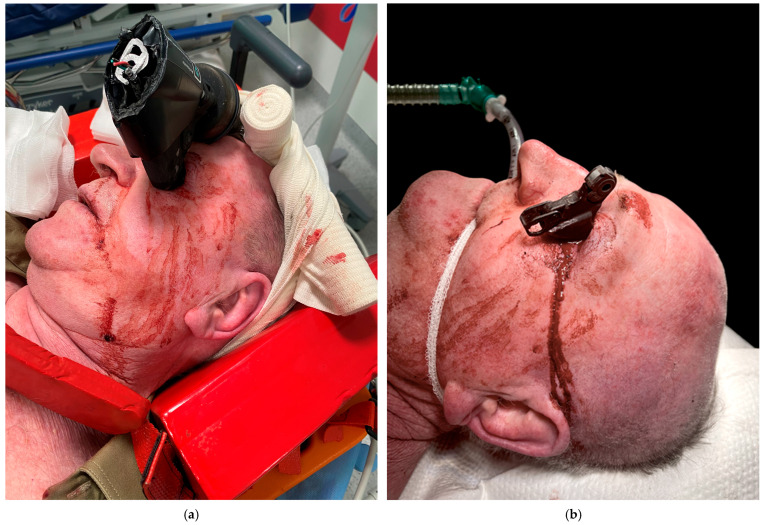
Orbital fractures represent up to 50% of all facial skeletal injuries and have recently emerged as a potential consequence of e-scooter–related accidents [1,2,3]. In contrast, penetrating head injuries are rare, accounting for approximately 0.4% of all head trauma cases, and are most commonly associated with falls, motor vehicle collisions, and explosions. Among these, transorbital penetrating injuries represent up to 24% of adult penetrating head injuries [4,5]. Isolated cases of penetrating intracranial injuries caused by bicycle brake handles have been reported in the literature, unfortunately, most with fatal outcomes [6,7,8]. A 76-year-old male patient was admitted to the emergency department with a transorbital intracranial injury caused by an e-scooter brake handle. The patient was not riding the e-scooter but sustained the injury after a same-level fall onto a shared electric scooter. On admission, he was conscious but intoxicated, with a blood alcohol concentration of 2.13‰ (according to the hospital laboratory), and had no recollection of the incident. (**a**) The fire brigade team cut the brake handle from the handlebar, and the remaining portion was stabilized with bandages for transport and for the CT-angiography (CTA) examination. (**b**) Prior to surgery, the brake handle required further dismantling to allow for craniotomy. The patient was transferred to the operating theatre. A hemi-coronal incision was made in the left frontal region, extending across the midline. A left pterional–parasagittal craniotomy was performed. The site of penetration of the foreign body through the orbital roof into the left frontal lobe was identified, and the foreign body was removed under direct visual control. Following removal, cerebrospinal fluid (CFS) outflow from the lateral ventricle was observed. Inspection of the surgical field revealed no evidence of bleeding, and the ventricle was closed using TachoSil. After removal of the foreign body, the left eyeball appeared intact, with a narrow and symmetrical pupil. The orbit was inspected through the wound in the upper eyelid. Apart from minor soft tissue injuries, no injury to the extraocular muscles was detected. Reconstruction of the anterior cranial fossa was then performed. The orbital roof was repositioned, and a “sandwich” dural repair was completed using TachoSil and tissue glue. The bone flap was reduced and secured with sutures. During hospitalization, the patient underwent endoscopic revision to assess for CSF leakage into the nasal cavity [9]. Due to the extent of brain injury, the patient subsequently required admission to the psychiatric ward.

**Figure 2:**
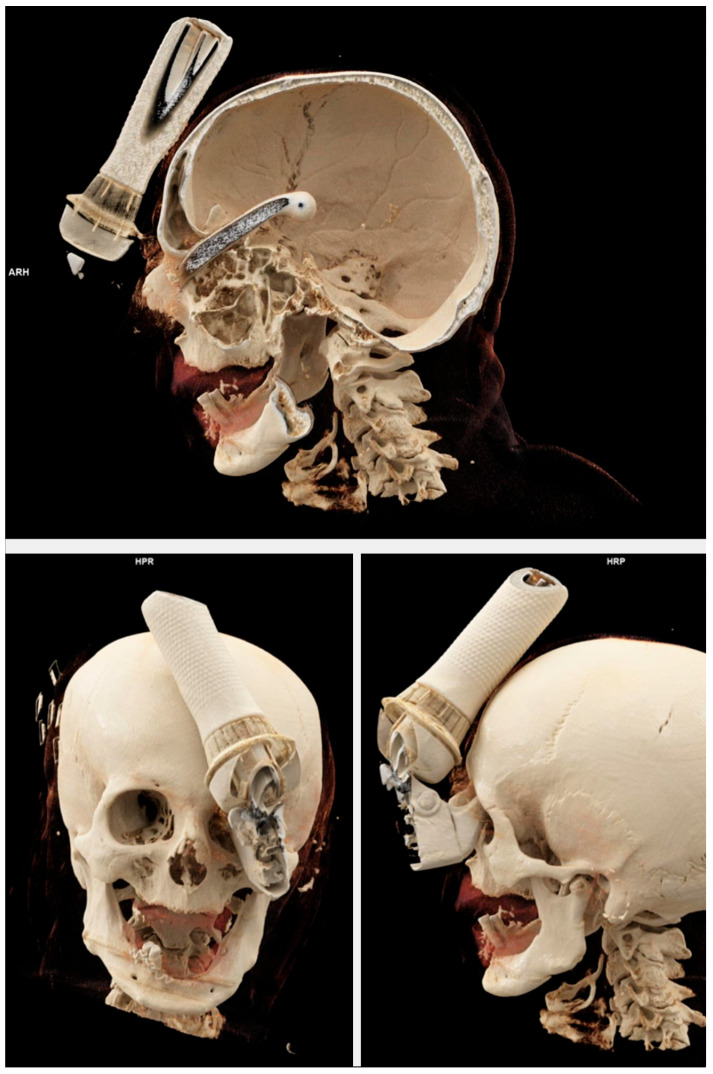
Three-dimensional bony reconstruction from CTA demonstrating a transorbital intracranial injury caused by an e-scooter brake handle.

**Figure 3:**
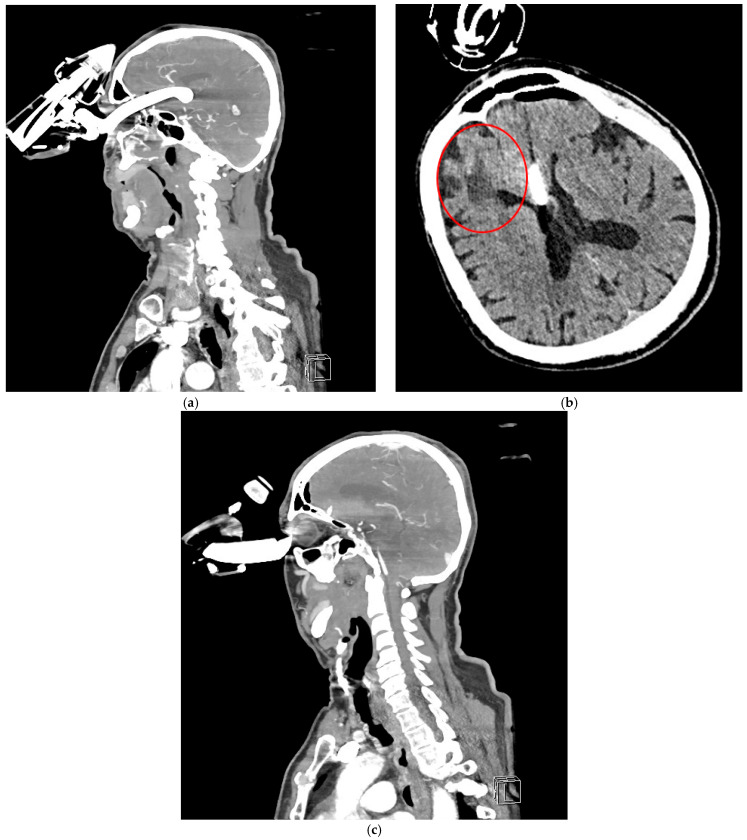
CTA revealed a foreign body penetrating the left orbit into the anterior cranial fossa, extending into the third ventricle to a depth of approximately 64 mm (**a**). The anterior cerebral arteries were visualized along the foreign body, both running on its left side. No significant intracranial hemorrhage was observed. Additionally, features of porencephaly were noted, likely resulting from a previous injury to the right frontal lobe (red circle—the patient had a history of two head CT scans at our hospital, in 2012 and 2014, which showed evidence of porencephaly) (**b**). The ventricular system was normal in size and position, without displacement or enlargement. (**c**) The left eyeball was displaced laterally and inferiorly, compressed by the foreign body, but showed no evidence of direct injury.

**Figure 4:**
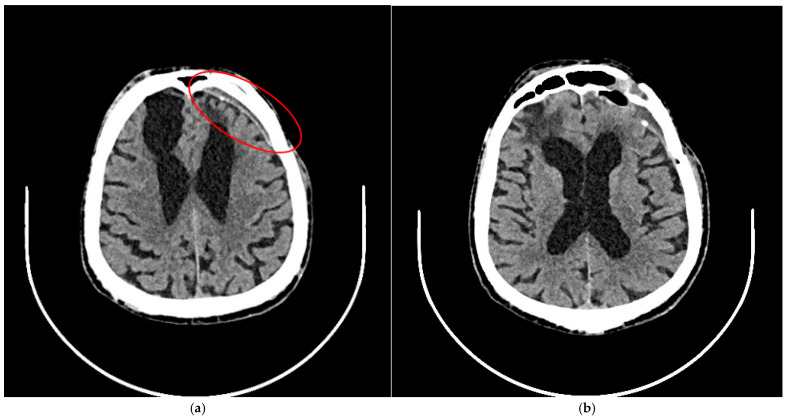
A follow-up CT scan six months after treatment showed no evidence of new intracranial hemorrhage. (**a**) A narrow chronic subdural hematoma was noted (red circle). (**b**) Extensive hypodense post-traumatic changes were observed at the base of both frontal lobes. The ventricular system was significantly enlarged and asymmetrical, with evidence of right-sided retraction. There was no displacement of midline structures.

**Figure 5:**
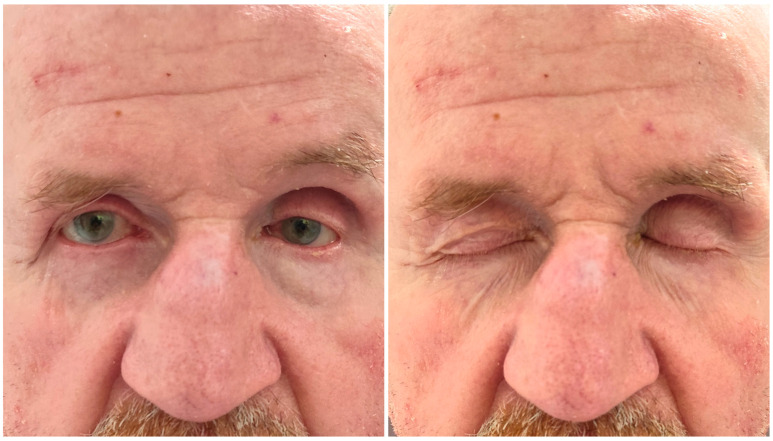
Clinical examination one year after the injury revealed atrophy of the orbital fat in the superior quadrants of the left orbit, accompanied by mild eyelid ptosis. The patient had a history of bilateral cataract surgery before injury. Visual acuity was 0.9 in the right eye (OD), measured without and with correction (NC/CC), and 0.32 in the left eye (OS) with correction using pinhole testing. Fundus examination revealed pink optic discs with clearly defined margins, maculae without reflex, and retinal vessels appropriate for age in both eyes. The retinas were attached in the areas accessible to examination. No diplopia was noted. Neurocognitive assessment revealed deficits primarily affecting auto- and allopsychic orientation, recent memory, auditory encoding of new information, and the selective retrieval of previously learned material from long-term memory. Memory gaps were frequently filled with confabulations. In addition, verbal fluency was impaired, likely secondary to executive dysfunction.

## Data Availability

No new data were created or analyzed in this study. Data sharing is not applicable to this article.
